# Initiation of and long-term adherence to secondary preventive drugs after acute myocardial infarction

**DOI:** 10.1186/s12872-016-0283-6

**Published:** 2016-05-31

**Authors:** Sigrun Halvorsen, Jarle Jortveit, Pål Hasvold, Marcus Thuresson, Erik Øie

**Affiliations:** Department of Cardiology, Oslo University Hospital Ulleval and University of Oslo, Postboks 4956, Nydalen, 0424 Oslo Norway; Department of Cardiology, Sørlandet Hospital, Arendal, Norway; AstraZeneca NordicBaltic, Södertälje, Sweden; Statisticon, Uppsala, Sweden; Department of Internal Medicine, Diakonhjemmet Hospital, and Center for Heart Failure Research, University of Oslo, Oslo, Norway

**Keywords:** Acute myocardial infarction, Secondary prevention, Medication adherence

## Abstract

**Background:**

Secondary preventive drug therapy following acute myocardial infarction (AMI) is recommended to reduce the risk of new cardiovascular events. The aim of this nationwide cohort study was to examine the initiation and long-term use of secondary preventive drugs after AMI.

**Methods:**

The prescription of drugs in 42,707 patients < 85 years discharged alive from hospital after AMI in 2009–2013 was retrieved by linkage of the Norwegian Patient Register, the Norwegian Prescription Database, and the Norwegian Cause of Death Registry. Patients were followed for up to 24 months.

**Results:**

The majority of patients were discharged on single or dual antiplatelet therapy (91 %), statins (90 %), beta-blockers (82 %), and angiotensin-converting enzyme inhibitors (ACEI)/angiotensin receptor II blockers (ARB) (60 %). Patients not undergoing percutaneous coronary intervention (PCI) (42 %) were less likely to be prescribed secondary preventive drugs compared with patients undergoing PCI. This was particular the case for dual antiplatelet therapy (43 % vs. 87 %). The adherence to prescribed drugs was high: 12 months after index AMI, 84 % of patients were still on aspirin, 84 % on statins, 77 % on beta-blockers and 57 % on ACEI/ARB. Few drug and dose adjustments were made during follow-up.

**Conclusion:**

Guideline-recommended secondary preventive drugs were prescribed to most patients discharged from hospital after AMI, but the percentage receiving such therapy was significantly lower in non-PCI patients. The long-time adherence was high, but few drug adjustments were performed during follow-up. More attention is needed to secondary preventive drug therapy in AMI patients not undergoing PCI.

**Electronic supplementary material:**

The online version of this article (doi:10.1186/s12872-016-0283-6) contains supplementary material, which is available to authorized users.

## Background

Ischemic heart disease is a common cause of death in industrialized countries and accounts for a large proportion of hospital admissions in Norway [1]. Approximately 13,000 men and women are diagnosed annually with acute myocardial infarction (AMI) [2]. Secondary preventive drug therapy, e.g. platelet inhibitors, statins, beta-blockers and angiotensin-converting enzyme inhibitors (ACEI)/angiotensin receptor II blockers (ARB), is recommended following AMI to reduce the risk of new cardiovascular events and death [3–7]. However, an underuse of secondary preventive drugs has previously been observed following AMI, especially in patients not undergoing percutaneous coronary intervention (PCI) [8]. Despite their elevated cardiovascular risk [9], still many AMI patients are not treated according to guidelines [10]. This may be related to under-prescription, reduced adherence, and/or under-dosing of secondary preventive drug therapy [11, 12]. A potential source for the underuse of recommended secondary preventive drugs could be the shift of treatment responsibility from the hospitals to the general practitioners in the primary care setting. The extent to which the hospital-initiated treatment is continued as initially prescribed, the doses adjusted or drugs switched to another type of drug within the same drug class, is not known. Comprehensive analyses of initiation and adherence in different patient populations are essential to improve long-term use of secondary preventive drugs and cardiovascular outcomes [13].

The aim of this nationwide cohort study was to examine the initiation and long-term use of secondary preventive drug in patients hospitalized with AMI in Norway during the years 2009 to 2013.

## Methods

### Data sources

This observational, historical cohort study was based on data from three Norwegian national registries: 1) The Norwegian Patient Register covering all hospital admissions and including diagnoses according to the International Classification of Diseases, 10th revision, Clinical Modification (ICD-10-CM) [14]; 2) the Norwegian Prescription Database registering all pharmacy dispenses [15]; and 3) the Norwegian Cause of Death Registry registering all deaths [16]. The prescription of drugs in patients discharged alive from hospital after AMI in 2009–2013 was retrieved by linkage of the Norwegian Patient Register, the Norwegian Prescription Database, and the Norwegian Cause of Death Registry. Patients were followed for up to 24 months. The Norwegian Institute of Public Health performed the data linkage. Data were anonymised before further analysis. The linked database was managed by The Norwegian University of Science and Technology, Trondheim, Norway.

### Study population

All patients below 85 years of age who were admitted to hospital with a primary diagnosis of AMI (index AMI) (ICD-10: I21) between 1 January 2009 and 30 November 2013 and alive 30 days after discharge were included in this study. Patients 85 years or older were excluded for two reasons: 1) their likelihood of long-term use of secondary preventive drugs might be extensively confounded by fragility; 2) older patients have an increased risk of long-term institutional stays where the drug use cannot be captured by the available registries. Patients were classified as PCI and non-PCI patients depending on whether PCI was performed or not up to 30 days after index-AMI. The study population was further stratified into two groups; ≤75 years and 76–84 years.

Index AMI was defined as the first recorded primary diagnosis of AMI for a patient during the specified time-period (not necessarily the patient’s first AMI). All residents in Norway are covered by a national health security system with a universal tax-funded access to primary and secondary health care, including secondary preventive drugs recommended after AMI.

### Follow-up

Observational data on drug prescriptions were collected up to 24 months following AMI, or until 31 December 2014 or death (whichever occurred first).

### Drug treatment and adherence

Drug treatment at discharge for index AMI were calculated from dispensed drugs from pharmacies one year within and until 30 days after the index AMI; either a prior dispensing covering day 0 to day 30 or a new dispensing within day 0 to day 30. Drug adherence was defined as the proportion of patients on the treatment of interest at each day from 12 months prior to the date of hospitalization for AMI until a maximum of 24 months after. The calculation of drug use (days on treatment) was based on the prescribed dose and on the number of pills collected or delivered from the pharmacies. Whether or not the pills were taken by the patients, were not assessed. If a patient had a gap in collection of drugs, the patient was defined as a non-user from last calculated day with available drug. Furthermore, if a patient after a gap, again collected the same drug from the pharmacy, the patient was defined as a user from that actual date, and if a patient was switched to another type of drug within the same drug class after the index AMI episode, the patient was defined as a user.

In the separate analysis of the adherence to the P_2_Y_12_-antagonists clopidogrel, prasugrel or ticagrelor during the first 18 months after AMI, the proportion of all patients still alive continuing on the same P_2_Y_12_ antagonist as at discharge was estimated.

In order to describe changes in drug treatment over time, treatment at discharge for index AMI was compared to treatment in the post AMI period (dispensed during 12˗18 months after the AMI).

### Statistical analyses

Data are presented as mean with standard deviation for continuous variables and absolute and relative frequencies for categorical variables. Patients were stratified by age (≤75 years or 75–84 years) and by PCI status (PCI or no PCI). Statistical analyses were performed using SAS version 9.3 (SAS Institute Inc, Cary, NC, USA) and R version 3.2.2 [17].

## Results

**Fig. 1:**
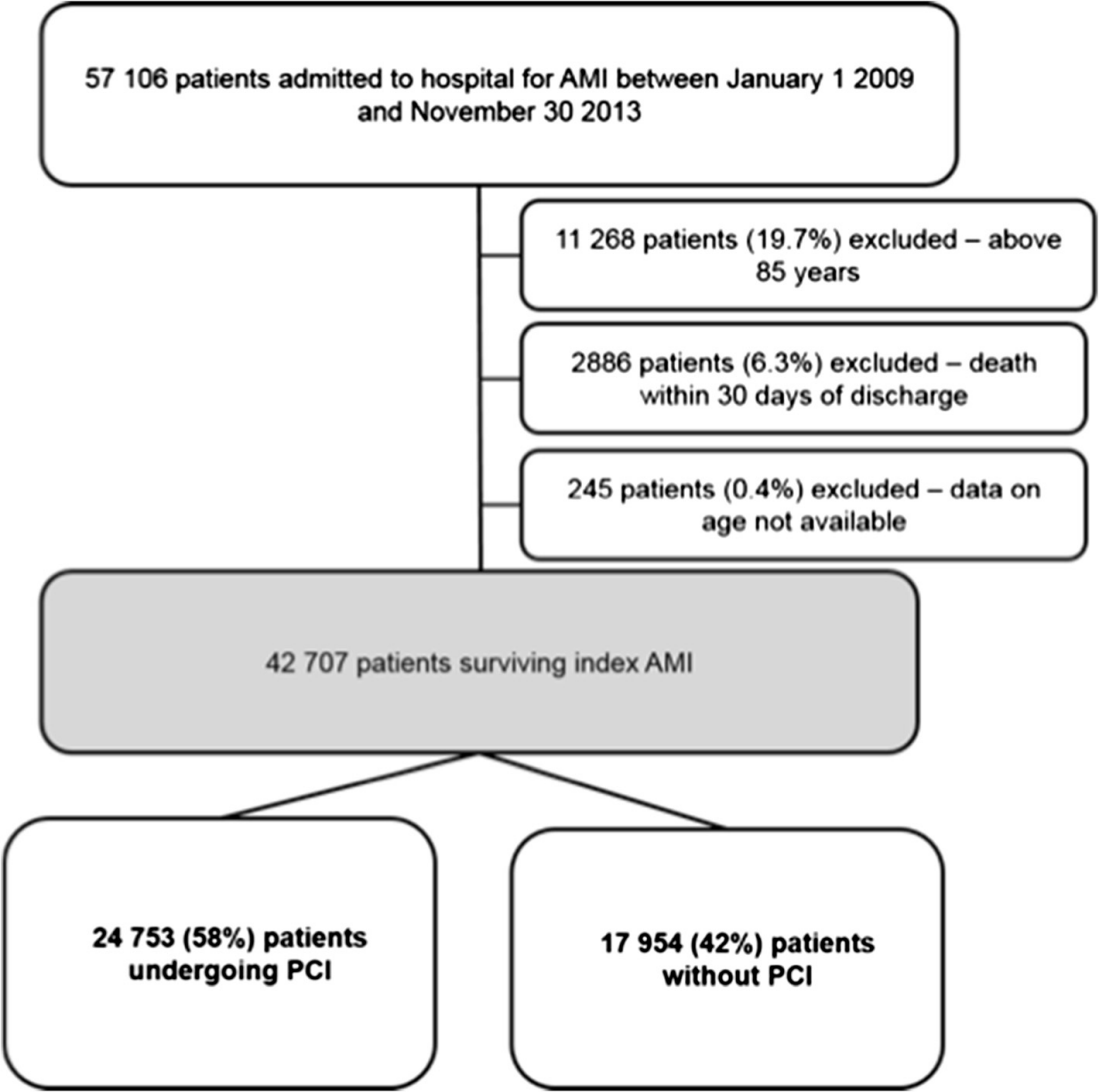
Flow chart of the study population

A total of 57,106 individuals were admitted to Norwegian hospitals for AMI during the study period, of whom 45,838 (80.3 %) were younger than 85 years. Of these, 42,707 (93.2 %) were alive 30 days after hospital discharge and could be included in the study (Fig. 1). Overall, 70 % of the patients were men and mean age was 65.8 years (standard deviation 11.8) (Table 1). A total of 58 % of the patients underwent PCI, with an increasing proportion during the study period (from 53 % to 63 %) (Additional file 1, Table 1). Patients undergoing PCI were younger and more often male compared with the medically treated patients (Table 1).

**Table 1:** Drug treatment at discharge after index AMI. Patients stratified by age (≤75 years or 75–84 years) and by PCI status (PCI or no PCI)

	Age ≤75 years		Age 75–84 years		Total
	*n* = 30 843		*n* = 11 864		*n* = 42 707
	PCI	No PCI	PCI	No PCI	
	*n* = 19 835	*n* = 11 008	*n* = 4918	*n* = 6946	
	(64.3)	(35.7)	(29.3)	(70.7)	
Women	4065 (20.5)	3478 (31.6)	1819 (37.0)	3357 (48.3)	12 719 (29.8)
Age, mean (SD)	59.8 (9.1)	61.6 (9.6)	79.0 (2.8)	80.0 (2.8)	65.8 (11.8)
DAPT	17 505 (88.3)	5107 (46.4)	3877 (78.8)	2597 (37.4)	29 086 (68.1)
Only P2Y_12_ inhibitor	668 (3.4)	354 (3.2)	202 (4.1)	295 (4.3)	1519 (3.6)
Only ASA	288 (1.5)	3105 (28.2)	151 (3.1)	1860 (26.7)	5404 (12.3)
Only OAC	21 (0.1)	312 (2.8)	15 (0.3)	339 (4.9)	687 (1.6)
OAC + DAPT	925 (4.7)	241 (2.2)	473 (9.6)	165 (2.4)	1804 (4.2)
OAC + P2Y_12_ inhibitor	58 (0.3)	40 (0.4)	28 (0.6)	58 (0.8)	184 (0.4)
OAC + ASA	43 (0.2)	520 (4.7))	32 (0.7)	529 (7.6)	1124 (2.6)
No antiplatelet or OAC	327 (1.7)	1329 (12.1)	140 (2.9)	1103 (15.9)	2899 (6.8)
Statins	19 168 (96.6)	9224 (83.8)	4574 (93.0)	5262 (75.7)	38 228 (89.5)
Beta-blockers	16 763 (84.5)	8529 (77.5)	4238 (86.2)	5456 (78.6)	34 986 (81.9)
ACE inhibitors	7977 (40.3)	3478 (31.7)	2027 (41.2)	2497 (36.0)	15 988 (37.4)
ARB	4822 (24.3)	2945 (26.8)	1597 (32.5)	2072 (29.8)	11 346 (26.8)

Numbers in parentheses are percentages of total number of patients in the group. *SD* denotes standard derivation, *DAPT* dual antiplatelet therapy, *AMI* Acute myocardial infaction, *ASA* Acetylsalicylic acid, *OAC* Oral anticoagulants, *ACE* Angiotensin-converting enzyme, *ARB* Angiotensin II receptor blocker

### Initiation of secondary preventive drugs

The prescription of secondary preventive drugs at discharge is shown in Tables 1 and 2. The majority of patients were discharged on single or dual antiplatelet therapy (DAPT) (19 % and 72 %, respectively), statins (90 %), beta-blockers (82 %), and ACEI/ARB (60 %). The percentage receiving these drugs were slightly lower in patients 75–84 years compared to patients ≤75 years, except for ACEI/ARB which was prescribed slightly more often in the elderly (Table 1).

**Table 2:** Secondary preventive drugs at discharge from hospital for index AMI and 12˗18 months later; patients <85 years

	Secondary preventive drugs at discharge for index AMI		Secondary preventive drugs 12–18 months after index AMI			
	(*n* = 42 707)		(*n* = 28 767)			
	*n* (%)	Mean dose (mg)	*n* (%)	Mean dose (mg)	*n* (%) switched to another drug within same drug class in post-AMI period	*n* (%) changed dose of actual drug in post-AMI period
*Statins*	38 228 (89.5)		24 062 (83.6)			
Simvastatin	23 528 (55.1)	37.2	12 478 (43.4)	38.4	2571 (15.7)	1415 (8.7)
Atorvastatin	17 084 (40.0)	56.9	10 913 (37.9)	52.1	592 (6)	1755 (17.8)
Rosuvastatin	225 (0.5)	17.7	367 (1.3)	19.6	10 (9.6)	13 (12.5)
Pravastatin	1158 (2.7)	33.5	651 (2.3)	33.5	120 (20.9)	39 (6.8)
*ACEI/ARB* ^a^	25 445 (59.6)		16 274 (56.6)			
Losartan	3658 (8.6)	66.6	2049 (7.1)	66.5	153 (6.9)	238 (10.8)
Candersartan	4157 (9.7)	13.1	2778 (9.7)	13.5	145 (5.4)	467 (17.4)
Valsartan	1503 (3.5)	124.9	865 (3.0)	125.2	68 (7.2)	127 (13.4)
Irbesartan	1530 (3.6)	235.6	770 (2.7)	237.3	72 (7.8)	61 (6.6)
Enalapril	2951 (6.9)	11.8	1657 (5.8)	11.9	170 (8.9)	324 (16.9)
Ramipril	11 492 (26.9)	3.5	6999 (24.3)	4.5	733 (8.8)	2156 (25.8)
Lisinopril	1753 (4.1)	11.3	949 (3.3)	11.3	121 (10.3)	207 (17.5)
*Beta-blockers*	34 986 (81.9)		22 061 (76.7)			
Metoprolol	30 874 (72.3)	60.6	18 920 (65.8)	62.3	702 (3.2)	4452 (20.3)
Atenolol	1381 (3.2)	52.4	457 (1.6)	51.0	117 (21.6)	54 (10)
Propranolol	305 (0.7)	66.2	82 (0.3)	80.4	26 (26.3)	6 (6.1)
Sotalol	358 (0.8)	80.3	138 (0.5)	75.9	49 (33.3)	14 (9.5)
Bisoprolol	2477 (5.8)	4.1	1856 (6.5)	4.4	64 (4)	310 (19.3)
Carvedilol	1298 (3.0)	15.8	813 (2.8)	15.6	73 (9.1)	123 (15.3)

Numbers in parentheses are percentages of total number of patients in the group

*ACEI* Angiotensin-converting enzyme inhibitor, *AMI* Aciute myocardial infraction, *ARB* Angiotensin II receptor blocker

^a^some patients were prescribed both ACEI and ARB

**Fig. 2:**
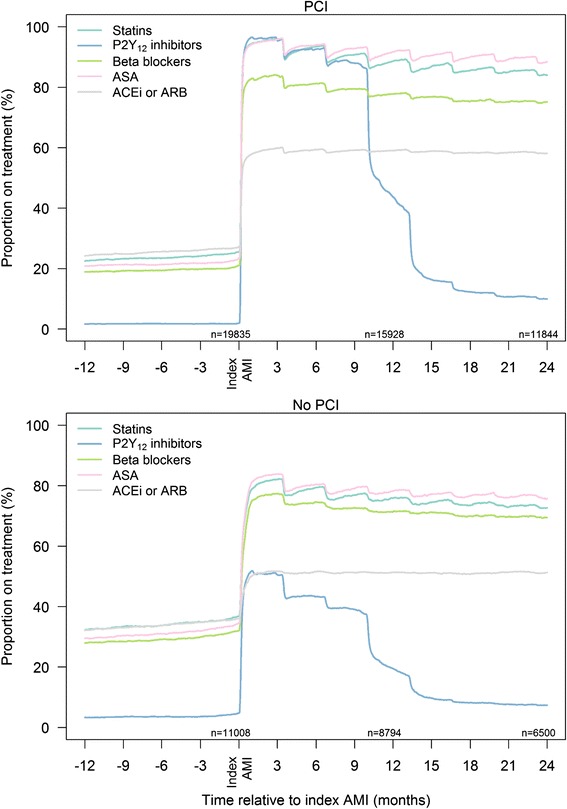
Adherence to secondary preventive drugs over time in AMI patients ≤75 years with or without PCI. Norway 2009–2013. *Abbreviations:* ASA, acetylsalicylic acid; ACEI, angiotensin-converting enzyme inhibitor; AMI, acute myocardial infarction; ARB, angiotensin II receptor blocker; PCI, percutaneous coronary intervention

**Fig. 3:**
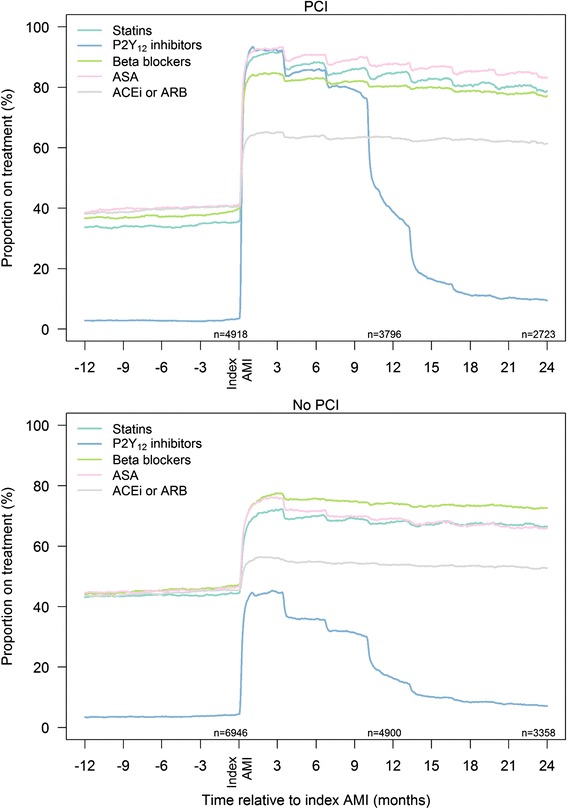
Adherence to secondary preventive drugs over time in AMI patients 76–84 years with and without PCI. Norway 2009–2013. *Abbreviations*: ASA, acetylsalicylic acid; ACEI, angiotensin-converting enzyme inhibitor; AMI, acute myocardial infarction; ARB, angiotensin II receptor blocker; PCI, percutaneous coronary intervention

Patients undergoing PCI were prescribed secondary preventive drug therapy more often than patients not undergoing PCI (Table 1). This was the case both for patients <75 years and patients 75–84 years. The difference in prescriptions was largest with respect to DAPT, which was prescribed in 92 % of the PCI patients vs. 45 % of patients not undergoing PCI (Table 1, Figs. 2 and 3). In contrast, non-PCI patients were prescribed other kinds of antithrombotic therapy more often than PCI patients: Aspirin monotherapy in 28 % vs. 2 %, oral anticoagulant (OAC) monotherapy in 4 % vs. 0 %, or OAC in combination with single antiplatelet therapy in 6 % vs. 1 %, respectively. However, 14 % of the non-PCI patients were discharged with neither antiplatelet drugs nor OAC, compared to 2 % of the PCI patients. Surprisingly, the differences in prescription pattern between PCI and non-PCI patients were found also with respect to other types of secondary preventive drugs (Table 1).

The mean dose of statins at discharge was 37 mg for simvastatin (55 % of patients) and 57 mg for atorvastatin (40 % of patients). The mean doses of ACEI/ARB and beta-blocker at discharge were 35-60 % and 30-50 % of maximal recommended doses, respectively (Table 2).

### Adherence to secondary preventive drugs

The overall long-time adherence was high among all patients initiated on treatment with statins, beta-blockers and ACEI/ARB (Figs. 2 and 3, Table 2). The proportions of patients using statins, ACEI/ARB and beta-blockers were reduced by 6 %, 3 % and 4 %, respectively, after one year. No major differences in drug adherence was observed between PCI and non-PCI patients, or between patients ≤75 and 76–84 years. When primary health care physicians took over the prescription responsibility for these patients (approximately 3 months after the AMI), no overall change in adherence was found. Approximately 20 % of patients changed to another drug within the same drug class, or changed the dose of statin, beta-blocker and ACEI/ARB within 12–18 months after the AMI (Table 2).

**Fig. 4:**
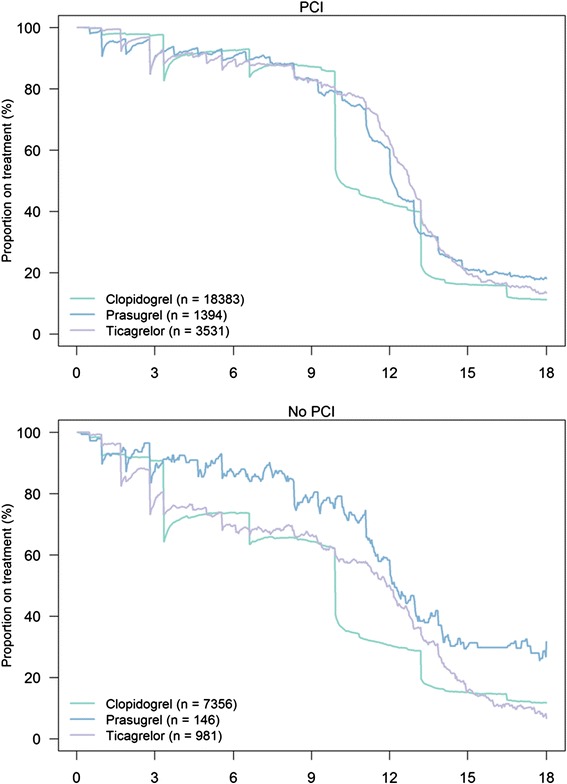
Adherence to treatment with different P2Y_12_ inhibitors in AMI patients <85 years with and without PCI. Norway 2009–2013. *Abbreviations*: AMI, acute myocardial infarction; PCI, percutaneous coronary intervention

The adherence to antiplatelet drugs was also high (Figs. 2 and 3). After 12 months, 84 % of patients were still on aspirin; 83 % after 18 months. The adherence to P_2_Y_12_ inhibitors are shown in more detail in Fig. 4 and Additional file 1: Table S2. Patients not undergoing PCI had a shorter length of time on treatment with a P_2_Y_12_ inhibitor compared with PCI patients, with a substantial proportion of these patients discontinuing treatment already after three months. The majority of the PCI patients treated with ticagrelor and prasugrel maintained the treatment through 12 months. Many PCI patients on clopidogrel discontinued P_2_Y_12_ inhibitor after nine months.

## Discussion

This nationwide observational cohort study, including all patients younger than 85 years surviving an AMI in Norway during the years 2009 to 2013, showed a generally high long-term adherence to the prescribed treatment with antiplatelet drugs, statins, beta-blockers and ACEI/ARB. The shift of responsibility for prescribing secondary preventive drugs from hospital to primary health care showed a sustained high use of the originally prescribed drugs. Only few patients switched to another type of statin or changed the dose of statin, beta-blocker and ACEI/ARB during the first two years after the AMI. For patients not undergoing PCI, a smaller proportion was discharged with secondary preventive drugs following their AMI compared with patients undergoing PCI, and less than half were prescribed DAPT at discharge.

Contemporary national level data describing long-term adherence to secondary preventive medications, i.e. antiplatelet drugs, statins, beta-blockers, and ACEI/ARB in AMI populations comparing younger vs. older and PCI vs. non-PCI patients are scarce. Furthermore, little is known on the impact of change in drug treatment responsibilities as AMI patients are transferred from hospital care to primary health care.

The initiation of secondary preventive drugs in our study was considerably higher than was found in a previous Danish nationwide study [12]. In patients admitted with a first AMI between 1995 and 2002 in Denmark, only 58 % received beta-blockers, 29 % ACE-inhibitors, and 34 % statins at discharge [12]. Although long-term compliance was reasonably good, patients who did not start treatment shortly after discharge had a low probability of starting treatment later [12]. In a more recent Swedish study, both initiation and long-term adherence to secondary preventive drug therapy was higher and similar to our findings: 82 % of AMI patients were still on aspirin 12 months post AMI, 73 % on statins and 80 % on beta-blockers [18]. A high degree of initiation of secondary preventive drug therapy was also observed in a recent study from the United States [13]. However, when patients were divided into risk groups based on the Global Registry of Acute Coronary Event (GRACE) risk score at hospital discharge, high-risk patients had a lower likelihood of receiving all appropriate therapies at discharge compared with low-risk patients.

Our data may indicate that if secondary preventive drug therapy after AMI is not prescribed at hospital discharge, the likelihood of receiving such treatment is limited. Thus, initiation of guideline-recommended secondary preventive drug therapy after AMI seems to depend on the hospital physicians, in accordance with the previous observations from Denmark [12]. The present study further demonstrates that only minor dose adjustments for drugs prescribed at discharge were performed by the primary care physicians during follow-up. Thus, Norwegian primary care physicians seemed reluctant to changing already prescribed secondary preventive drug therapies after AMI.

This finding further underscores the importance of drug prescription prior to discharge of AMI patients from hospitals. Not only should AMI patients be prescribed guideline-recommended secondary preventive drugs, but the hospital physicians could also advice on further up-titration and the target doses of the prescribed drugs in the discharge summary sent to the patient’s general practitioner. It is also important that the general practitioners are updated on current guidelines and take the responsibility for optimizing secondary preventive treatment after AMI. Interestingly, patients not undergoing PCI were less likely to receive guideline-recommended secondary preventive drugs compared with patients undergoing PCI. For example, a 10–20 % higher use of statins and beta-blockers was seen in PCI patients vs. patients not undergoing PCI. Furthermore, a larger portion of patients not undergoing PCI was discharged without a P2Y_12_ inhibitor (48 % and 55 %, respectively, in patients ≤75 or 76–84 years) compared with PCI patients (4 % and 7 %). The reasons for this undertreatment of non-PCI patients are not known. We might speculate that a higher degree of comorbidities might be present in these patients, making the physicians more selective in their prescription of secondary preventive drugs.

The mean doses of both ACEI, ARB and beta-blockers in our study were lower than the target doses in randomized trials studying the efficacy and safety of these drugs. The reasons for the choice of these lower doses, or the lack of up-titration of doses after hospital discharge, are unknown. These drugs are used for a variety of indications, and since we have limited data on weight, blood pressure, heart rate, ejection fraction, comorbidities and on the specific indications for the various drugs (e.g. for ACEI/ARB), we find it difficult to draw any firm conclusions regarding target doses and whether the doses used in our study were too low or not. With respect to statin treatment, it should be noted that during most of our study period, no specific statin dose was recommended and statins were prescribed mainly according to LDL-cholesterol levels.

We observed a generally short duration of P_2_Y_12_ inhibitor treatment for patients not undergoing PCI (Fig. 4), with a significant proportion of patients treated for only three months as also observed in a recent study from Sweden [19]. One possible reason for the longer treatment duration after PCI may be that P2Y_12_ inhibition is regarded particularly important after stent implantation to avoid stent thrombosis. However, ESC guidelines recommend P_2_Y_12_ inhibition for 12 months in all ACS patients [3–6].

A large proportion of patients discontinued P_2_Y_12_ inhibitor after nine months; mainly patients on clopidogrel treatment. One likely explanation to this finding was the nine months’ limited reimbursement of clopidogrel in Norway until September 2011 [20]. Prasugrel and ticagrelor have not had such reimbursement limitations. This finding demonstrates the influence the health authorities have on medical treatment by determining the prescription rules for reimbursements of various drugs.

### Limitations

Our data set provides a unique possibility to examine adherence to antiplatelet therapy, statins, beta-blockers and ACEI/ARB. It includes nationwide data from all patients hospitalized in Norway for AMI in 2009–2013, allowing analyses of a complete and unselected cohort of patients, and also allowing differentiation between younger and older patients and between patients undergoing PCI or not. This reduces potential problems arising from selection bias due to inclusion of selected hospitals, regions, or health care insurance systems. Furthermore, by restricting the inclusion to patients below 85 years of age, this study focuses on patients who normally would be considered to be treated according to guidelines. However, this register-based analysis also has certain limitations. As our analysis relied on ICD-10 codes, the possibility of coding errors cannot be ruled out, although the primary diagnoses of AMI previously have been shown to have high sensitivity and specificity [21]. A further subclassification of patients into those presenting with and without persistent ST-segment elevation on the electrocardiogram could not be performed due to non-validated ICD-10 coding specification at this level.

Further, due the study aim describing secondary drug adherence in a nationwide patient population, patients were included based on their first AMI during the observational period, i.e. not necessarily their first time AMI. Thus, the patient population changed during the inclusion period. While patients included early in the inclusion period may have had a recent history of AMI, patients included towards the end of the study period had to be event-free for a longer time. Following from this study design, the yearly numbers of included AMIs in our study decreased during the observational period (Additional file 1, Table 1). Furthermore, how a recent prior AMI episode would affect selection for invasive treatment and secondary prevention drugs is difficult to predict, but it cannot be excluded that these patients would receive a higher attention, and thus a higher likelihood of receiving guideline-recommended treatment.

The registry data were collected for administrative purposes and we did not have any information on smoking patterns, weight, blood pressure, laboratory data or socioeconomic status. Furthermore, the Norwegian Patient Register has only had nationwide coverage since 2009 and medical history from previous hospitalizations was not available.

## Conclusions

This nationwide observational study, including all patients in Norway below 85 years of age being alive 30 days after AMI during the years 2009 to 2013, showed a generally high long-term adherence to antiplatelet therapy, as well as treatment with statins, beta-blockers and ACEI/ARB. To a large extent, PCI patients received guideline-recommended treatment with secondary preventive drugs. Patients not undergoing PCI were less likely to be discharged with the recommended drugs. The shift of responsibility for prescribing drug treatment from hospital to primary health care did not to any major extent alter the already prescribed treatments. Thus, the majority of AMI patients remained on the secondary preventive treatment originally prescribed, further underlining the importance of prescribing guideline-recommended drug treatment at hospital discharge, and preferably including specialist guidance on future target doses in the discharge summary. The present study also indicates a need for more careful attention to secondary preventive drug therapy in AMI patients not undergoing PCI.

## Abbreviations

ACEI, angiotensin-converting enzyme inhibitor; AMI, acute myocardial infarction; ARB, angiotensin II receptor blocker; ASA, acetylsalicylic acid; DAPT, dual antiplatelet therapy; ICD-10-CM, International Classification of Diseases, 10th revision, Clinical Modification; OAC, oral anticoagulant; PCI, percutaneous coronary intervention.

## Additional file

Additional file 1: Table S1. Proportion of AMI patients undergoing PCI between 2009 and November 2013. **Table S2.** Adherence to P_2_Y_12_ inhibitors after index AMI. (DOCX 23 kb)
