# Supplementary material for: Initiation of and long-term adherence to secondary preventive drugs after acute myocardial infarction
Source: BMC Cardiovasc Disord. 2016 May 31;16:115. doi: 10.1186/s12872-016-0283-6 (PMC4886431; doi:10.1186/s12872-016-0283-6)
Supplement: Additional file 1: Table S1. — Proportion of AMI patients undergoing PCI between 2009 and November 2013. Table S2. Adherence to P2Y12 inhibitors after index AMI. (DOCX 23 kb) [file 12872_2016_283_MOESM1_ESM.docx]

**Supplementary data**

**Table 1.** **Proportion of AMI patients undergoing PCI between 2009 and November 2013**

|  | **2009**  n=9550 | **2010**  n=9084 | **2011**  n=8464 | **2012**  n=8317 | **2013**  n=7292 | **Total**  n=42 707 |
| --- | --- | --- | --- | --- | --- | --- |
| **PCI, n (%)** | 5061  (53.0) | 5122  (56.4) | 5048  (59.6) | 4948  (59.5) | 4574  (62.7) | 24 753  (58.0) |

**Table 2.** **Adherence to P_2_Y_12_ inhibitors after index AMI**

A. Number and proportion of PCI patients on P_2_Y_12_ inhibitors during follow-up

|  | **3 months** | **6 months** | **9 months** | **12 months** | **18 months** |
| --- | --- | --- | --- | --- | --- |
| **Clopidogrel, n (%)*** | 17632 (97.5%) | 16393 (92.6%) | 15061 (87.2%) | 7097 (42.6%) | 1742 (11.2%) |
| **Ticagrelor, n (%)*** | 2709 (89.7%) | 2064 (89.5%) | 1355 (82.9%) | 711 (63.2%) | 41 (13.4%) |
| **Prasugrel, n (%)*** | 1146 (90.1%) | 998 (90.3%) | 751 (83.1%) | 429 (60.3%) | 63 (18.1%) |

* The proportion of all patients still alive continuing on the same P_2_Y_12_ antagonist as at discharge

B. Number and proportion of non-PCI patients on P_2_Y_12_ inhibitors during follow-up

|  | **3 months** | **6 months** | **9 months** | **12 months** | **18 months** |
| --- | --- | --- | --- | --- | --- |
| **Clopidogrel, n (%)*** | 6412 (90.8%) | 4999 (73.8%) | 4183 (64.4%) | 1889 (30.5%) | 655 (11.8%) |
| **Ticagrelor, n (%)*** | 642 (77.5%) | 447 (69.6%) | 306 (66.4%) | 158 (50.0%) | 4 (6.7%) |
| **Prasugrel, n (%)*** | 122 (87.1%) | 108 (86.4%) | 93 (78.8%) | 60 (58.3%) | 13 (31.7%) |

* The proportion of all patients still alive continuing on the same P_2_Y_12_ antagonist as at discharge
